# *Clostridium perfringens* Epsilon Toxin Increases the Small Intestinal Permeability in Mice and Rats

**DOI:** 10.1371/journal.pone.0007065

**Published:** 2009-09-18

**Authors:** Jorge Goldstein, Winston E. Morris, César Fabián Loidl, Carla Tironi-Farinatti, Bruce A. McClane, Francisco A. Uzal, Mariano E. Fernandez Miyakawa

**Affiliations:** 1 Laboratorio de Fisiopatogenia, Departamento de Fisiología, Facultad de Medicina, Universidad de Buenos Aires, Buenos Aires, Argentina; 2 Instituto de Patobiología, Centro Nacional de Investigaciones Agropecuarias, Instituto Nacional de Tecnología Agropecuaria, Castelar, Buenos Aires, Argentina; 3 Instituto de Biología Celular y Neurociencia “Prof. E. De Robertis”, Facultad de Medicina, Universidad de Buenos Aires, Buenos Aires, Argentina; 4 Department of Molecular Genetics and Biochemistry, University of Pittsburgh School of Medicine, Pittsburgh, Pennsylvania, United States of America; 5 California Animal Health and Food Safety Laboratory System, San Bernardino Branch, School of Veterinary Medicine, University of California Davis, San Bernardino, California, United States of America; Charité-Universitätsmedizin Berlin, Germany

## Abstract

Epsilon toxin is a potent neurotoxin produced by *Clostridium perfringens* types B and D, an anaerobic bacterium that causes enterotoxaemia in ruminants. In the affected animal, it causes oedema of the lungs and brain by damaging the endothelial cells, inducing physiological and morphological changes. Although it is believed to compromise the intestinal barrier, thus entering the gut vasculature, little is known about the mechanism underlying this process. This study characterizes the effects of epsilon toxin on fluid transport and bioelectrical parameters in the small intestine of mice and rats. The enteropooling and the intestinal loop tests, together with the single-pass perfusion assay and *in vitro* and *ex vivo* analysis in Ussing's chamber, were all used in combination with histological and ultrastructural analysis of mice and rat small intestine, challenged with or without *C. perfringens* epsilon toxin. Luminal epsilon toxin induced a time and concentration dependent intestinal fluid accumulation and fall of the transepithelial resistance. Although no evident histological changes were observed, opening of the mucosa tight junction in combination with apoptotic changes in the lamina propria were seen with transmission electron microscopy. These results indicate that *C. perfringens* epsilon toxin alters the intestinal permeability, predominantly by opening the mucosa tight junction, increasing its permeability to macromolecules, and inducing further degenerative changes in the lamina propria of the bowel.

## Introduction

Epsilon toxin (ETX) produced by *Clostridium perfringens* types B and D is responsible for a highly fatal enterotoxaemia in livestock [1]. This toxin is secreted in the gut lumen as a prototoxin which then becomes fully active by the action of either the host's intestinal trypsin or a *C. perfringens* metalloproteinase [2]. Once fully activated, ETX is absorbed and spreads through the blood-stream, affecting the lungs, kidneys and the brain [3]. Sheep and goats suffering from *C. perfringens* type D enterotoxaemia can experience a disease ranging from a peracute form, with neurological signs and sudden death, to a chronic intestinal disease, including hemorrhagic diarrhea and colitis [1].

The disease associated with *C. perfringens* type D in young lambs is very brief, often less than 2 hours, with many lambs being found dead without premonitory signs, or dying after a few minutes of violent convulsive activity [4]. In lambs and goats inoculated intraduodenally with *C. perfringens* type D culture supernatant containing ETX, nervous signs or death were observed as soon as 30 minutes after inoculation [5]. Similarly, in mice, lethal effects were observed 2 hours after oral administration of ETX [6]. Although this evidence indicates that ETX is promptly absorbed from the gut lumen into the blood circulation, the mechanism involving this process is yet unknown.

Experimentally, necrosis of the colonic epithelium is observed in ETX treated tissues of sheep and goats [7]. This morphological damage can alter the function of the epithelial barrier, allowing toxin absorption through the large intestine. However, evident epithelial damage in the large intestine is rarely seen in natural cases of acute and peracute enterotoxaemia of sheep, thus suggesting that other segments of the gastrointestinal tract are involved in ETX absorption [1]. In fact, Losada-Eaton et al. [8] showed that ETX can be absorbed from both, the large and small intestines of experimentally inoculated mice.

Bullen and Batty [9] reported that filtrates containing ETX, increased immunoglobulin absorption in the intestine of mice and sheep and Fernandez-Miyakawa et al. [10] observed that pefringolysin-O, a 54 KDa thiol-activated hemolysin from *C. perfringens*, was only absorbed from the intestinal tract of mice when ETX was present, but in the absence of histological damage to the intestine. These results suggest that ETX affects the small intestinal permeability by a mechanism independent on histological damage.

ETX has the ability to induce water accumulation in the small intestine of sheep and goats [7]. This fluid imbalance may explain the diarrhea or watery intestinal contents observed in *C. perfringens* type D enterotoxaemia, particularly if the animals survive longer than a few hours [4], [11]. However, the physio-pathological mechanisms of fluid imbalance induced by ETX in the small intestine are unknown [12]. An augmented paracellular permeability of the small intestine could be responsible not only for the toxin absorption; it could be responsible, at least in part, for the fluid accumulation observed in the small intestine of sheep and goats. However, the available data to support this hypothesis is not only scanty but indirect.

In an attempt to address these issues, the aims of the present study were as follows: (i) to assess whether ETX can induce changes in the fluid transport of the mouse small intestine (ii) to investigate the effects of ETX on the electrophysiological parameters of the small intestine *in vitro* and *ex vivo*, and (iii) to determine whether ETX-induced intestinal changes alters epithelial permeability.

## Results

### Effects of ETX on intestinal fluid

**Figure 1:**
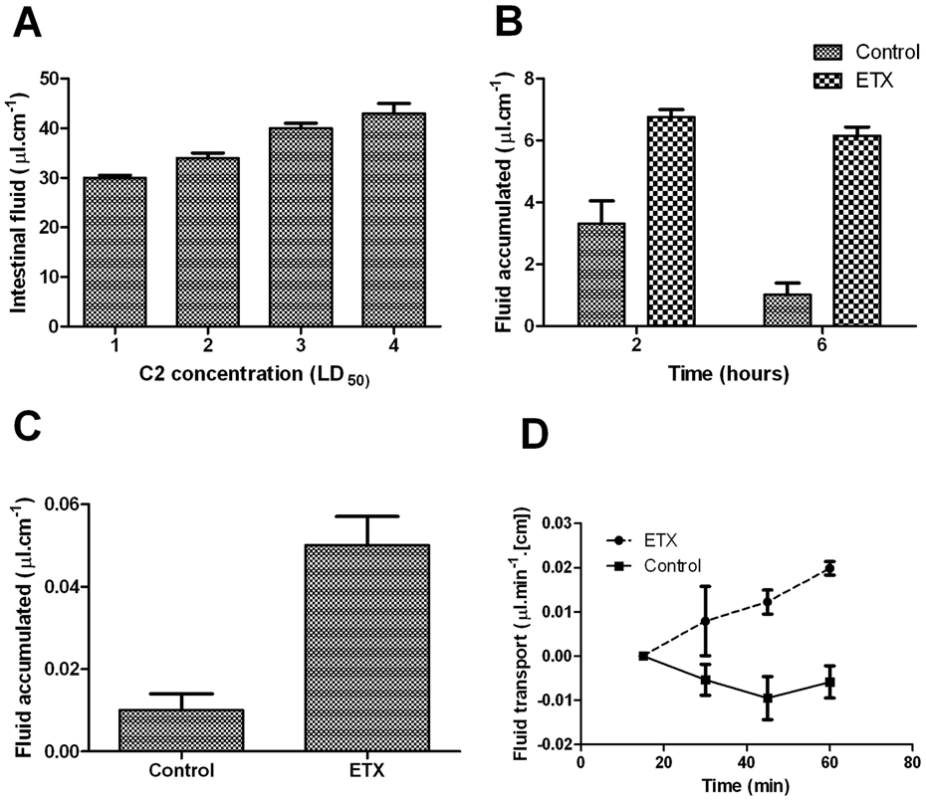
*C. perfringens* epsilon toxin alters fluid homeostasis in the small intestine. (A) The enteropooling assay detected small intestinal fluid accumulation induced by luminal enterotoxin. Groups of 4 mice were orally administered with different doses of *C. botulinum* C2 toxin and intestinal fluid was determined 6 hours after oral administration. The results shown are the mean±standard error of the mean. (B) Epsilon toxin of *C. perfringens* altered fluid homeostasis in the mouse small intestine. Enteropooling was measured in groups of 4 mice treated with toxin (1,000 LD_50_) or vehicle solution 2 and 6 h after oral administration. The results are expressed as the mean±standard error of the mean. (C) Epsilon toxin of *C. perfringens* produced fluid accumulation in mouse intestinal loops. Vehicle solution with or without 1,000 LD_50_ of toxin were injected in ligated ileal segments. The loops were excised 3 hours after injection of the toxin and intestinal water was determined gravimetrically. Each point represents the mean±standard error. (D) Basal absorption of water by the mouse small intestine was diminished by *C. perfringens* epsilon toxin when measured by the single-pass perfusion method. Perfusion began with an equilibration period of 45 min. Then, Ringer solution containing 500 LD_50_/ml of epsilon toxin or Ringer solution alone was perfused and after 60 minutes four 15-min samples were collected. The volume of each sample was determined. Each mouse was killed at the end of the perfusion, and the segment of jejunum was removed and its length measured. The absorbed or secreted volume was calculated as collected volume minus perfused volume and expressed per centimeter of perfused bowel per minute. Data were relativized to the value obtained at time 15. Changes become statistically significant after 45 minutes of toxin delivery (*P*<0.05).

Intestinal fluid accumulation in the mouse small intestine was initially determined by the enteropooling assay. Figure 1A shows an experiment carried out to confirm the ability of the enteropooling assay to evaluate enterotoxicity with *C. botulinum* C2 toxin, used as a validated intestinal control model. The results of this experiment show that *C. botulinum* C2 toxin, which had been previously described to have enterotoxic effects in the intestine of mice, produced fluid accumulation dependent of the toxin concentration 6 hours after the toxin was orally administered.

**Figure 2:**
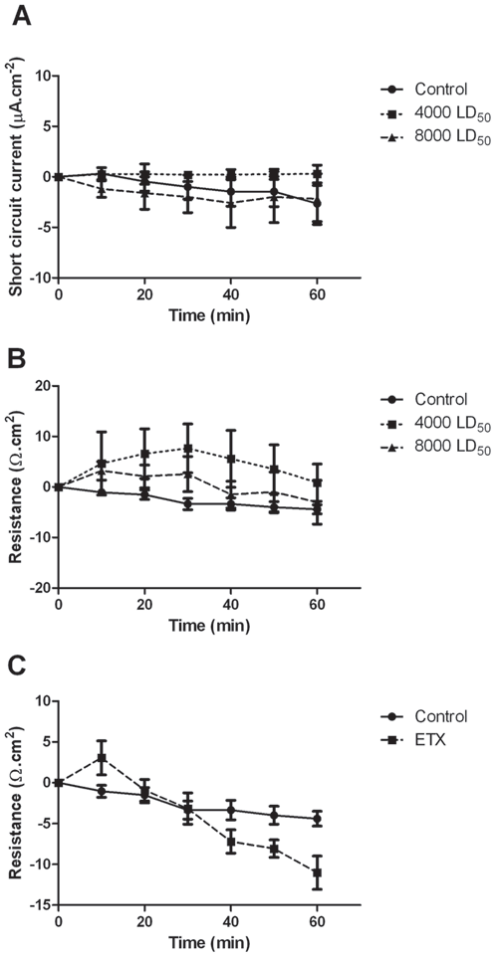
*In vitro* characterization of the effects of *C. perfringens* epsilon toxin in the electrical parameters of the murine small intestine. Epsilon toxin was incubated in the mucosal side of ileal sheets mounted in modified Ussing chambers. (A) Short circuit current (*I_sc_*) and (B) resistance (*Rt*) parameters were recorded each 10 minutes in tissues from 5 mice. (C) *Rt* values of ileal sheets incubated in the serosal side with 8,000 LD_50_/ml of epsilon toxin. Each bar represents results for 4 mice. Results are expressed as means±SEM.

The effects of ETX in the intestine of mice were then evaluated following the same experimental protocol. Figure 1B shows that intestinal fluid values was about 3 times greater than control 6 h after toxin delivery (Fig. 2, *P*<0.05). This increased fluid accumulation was observed as soon as two hour after toxin ingestion (Fig. 1B, *P*<0.05) and this effect was dependent of toxin concentration (Table 1). These ETX effects in the fluid transport were confirmed when 1,000 lethal dose fifity (LD_50_)/ml were incubated in ligated ileal loops during 3 h (Fig. 1C, *P*<0.05). Also, the water transport was affected in jejunum when the intestinal segments were incubated with 500 LD_50_/ml and changes were measured by the single-pass perfusion technique (Fig. 1D). Changes became statistically significant after 45 minutes of toxin delivery (*P*<0.05).

**Table 1:** Enteropooling of mice inoculated intragastrically with different ETX concentrations.

Epsilon toxin Dose (LD_50_)	Fluid accumulated (mg/cm)	Dried intestine weight (mg)	Intestinal length (cm)
0	28.4±1.6	272±10	36.13±0.89
125	32.7±0.4	276±5	32.38±1.66
500	36.5±2.9	279±8	31.11±0.68
2000	38.1±0.7	266±25	29.21±0.74

Six mice per treatment were inoculated intragastrically with ETX diluted in 1.5% PBS NaHCO_3_ solution. Mice were monitored during 3 hours and euthanized at the end of the experiments. Enteropooling was assessed after removal of the small intestine and determination of wet and dried weight. Final values were relativized to the intestinal length and expressed as mg/cm.

The effects of systemic ETX in the enteropooling assay were further studied. ETX was administered intravenously in mice and enteropooling was evaluated 3 hours later. A reduction in fluid accumulation dependent of the toxin concentration was observed (Table 2). Although the small intestine length was reduced when ETX was administered orally, this effect was not observed when this toxin was injected systemically (Table 1 and 2).

**Table 2:** Effect of ETX injected intravenously in mice in the enteropooling values.

Epsilon toxin IV Dose (LD50)	Fluid accumulated (mg/cm)	Dried intestine weight (mg)	Intestinal length (cm)
0	29.1±1.2	287±30	32.33±1.17
0.5	25.6±2.3	273±60	31.33±1.57
4	22.2±0.8	288±32	35.58±1.50

Six mice per treatment were injected intravenously with ETX diluted in peptone water. Mice were monitored during 1 hour and euthanized at the end of the experiments. Enteropooling was assessed after remotion of the small intestine and determination of wet and dried weight. Final values were relativized to the intestinal length and expressed as mg/cm.

### *In vitro* effects of ETX on the electrical parameters of mouse ileum

Ileal segments of mice mounted in Ussing-type chambers were incubated with ETX. The basal electrical parameters were not altered when concentrations up to 8,000 LD_50_/mL were incubated *in vitro* during 60 minutes in the mucosal side of the intestinal tissues (Fig. 2A and 2B). However, when 30 LD_50_ of ETX were incubated in the serosal side, a statistically significant decrease of the transepithelial resistance (Fig. 2C) was observed after 40 minutes of incubation (*P*<0.05).

Addition of glucose at the end of the experiments showed equivalent responses in treated and untreated tissues, indicating that glucose-Na^+^ active cotransport function was unaffected.

### *Ex vivo* effects of ETX on the electrical parameters of mouse ileum

**Figure 3:**
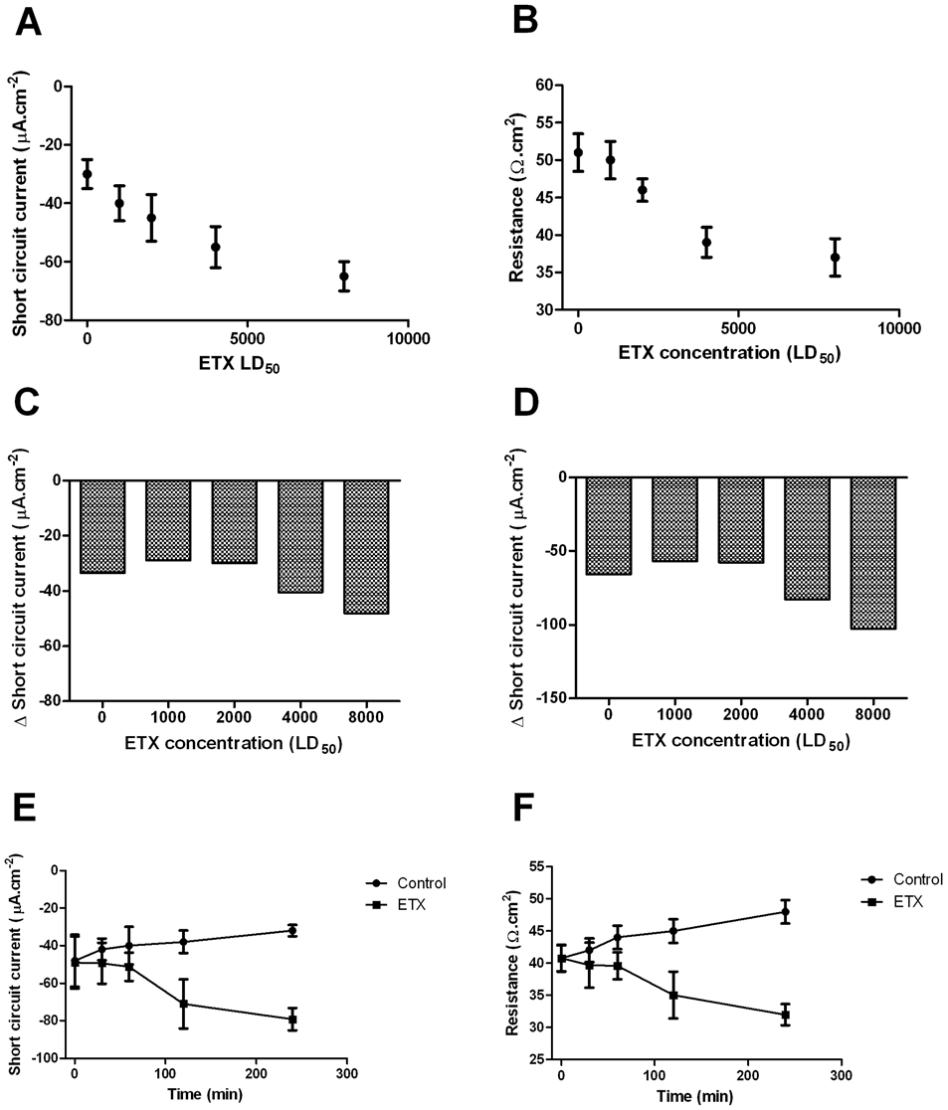
*Ex vivo* characterization of the effects of *C. perfringens* epsilon toxin in the electrical parameters of the murine small intestine. Intestinal loops were performed in groups of 4–6 anesthetized mice and injected with different concentrations of epsilon toxin in Ringer solution. In a particular set of experiments, intestines were removed after 3 hours of toxin injection and samples were mounted in Ussing chamber to measure changes in (A) short circuit current (*I_sc_*) and (B) resistance (*Rt*). Those tissues were exposed to (C) luminal glucose or (D) serosal teophylline and the *I_sc_* changes were recorded. In another set of experiments, ileal loops were injected with 1,000 LD_50_ of epsilon toxin and incubated during different periods of time. Values of (E) *I_sc_* or (F) *Rt* were recorded for those intestinal samples. Data are expressed as mean±SEM.

Segments of ileal loops treated during 3 h with different concentrations of ETX and then mounted *ex-vivo* in a modified Ussing chamber showed alterations of PD, *I_sc_* and Rt. A slight increase of *I_sc_* was observed when 1,000 LD_50_ or 2,000 LD_50_ were inoculated in ileal loops; those changes became statistically significant with 4,000 LD_50_ or 8,000 LD_50_ (Fig. 3A, *P*<0.05). A drop of Rt was evident when 2,000 LD_50_ or higher toxin concentrations were used (Fig. 3B, *P*<0.05). Glucose was added to the mucosal side after the electrical parameters were stabilized. An increase of glucose induced-*I_sc_* was observed at higher ETX doses (Fig. 3C). Theophylline stimulated the *I_sc_* when it was applied in the serosal side (Fig. 3D).

The same *ex-vivo* protocol was used with ileal loops incubated with 1,000 LD_50_ of ETX during different periods of time. An increase of *I_sc_* was observed in loops incubated with ETX at 120 or 240 minutes (Fig. 3E, *P*<0.05). The Rt was significantly reduced after 120 and 240 minutes of toxin incubation but changes were not observed with lower incubation times (Fig. 3F, *P*<0.05). An increase of glucose induced-*I_sc_* was also observed at 240 minutes when compared to control tisuses (Δ*I_sc_*, 30±10 vs 60±10 µAmp.cm^2^, *P*<0.05). ETX did not produce any significant alteration of the electrical parameter in the ileum 60 minutes after 4 LD_50_ were injected intravenously.

### Dilution potential

These so called dilution potentials are indicators for the ion selectivity of the epithelium and changes in these transepithelial potential differences reflect changes in ion selectivity of the paracellular pathway if the ion permeability of the cell membranes remains unaltered. In order to have information about the permeability of the paracellular pathway we measured the dilution potential generated when one half of the NaCl in the serosal solution was substituted with an equimolar amount of mannitol. This flux produced an increase of the dilution potential difference in luminal ETX treated ileum (4,000 LD_50_), indicating that the paracellular pathway has a different permeability to Na^+^ and Cl^−^, the permeability of the cation being greater than anion permeability. The magnitudes of dilution potential were 4.7±0.2 mV for control group and 5.8±0.4 mV for ETX group.

### ETX effects on electrical parameters of rat ileum

Basal electrical parameters of ileal segments of rats mounted in Ussing-type chambers were unaffected by concentrations of toxin up to 8,000 LD_50_/mL incubated in the mucosal side for at least 60 minutes. Segments of ileal loops treated with 4,000 LD_50_ of luminal ETX during 3 h and then mounted *ex-vivo* in a modified Ussing chamber showed alterations of *I_sc_* when compared to control loops (43.5±5.3y vs 56.2±5.6) and Rt (67.1±2.6 vs 54.5±5.5).

### Histological analysis

Although no major histological changes were observed in either control or epsilon treated loops, in some loops (control and ETX), mild polymorphonuclear infiltrate was observed in the submucosa and lamina propria, together with mild shortening of the villi.

Mild oedema was observed in the lamina propria of some ETX treated loops together with some degenerative cells, exhibiting pycnotic nuclei. These changes were not observed in the control tissues.

### Mucosal binding of ETX in the small intestine

**Figure 4:**
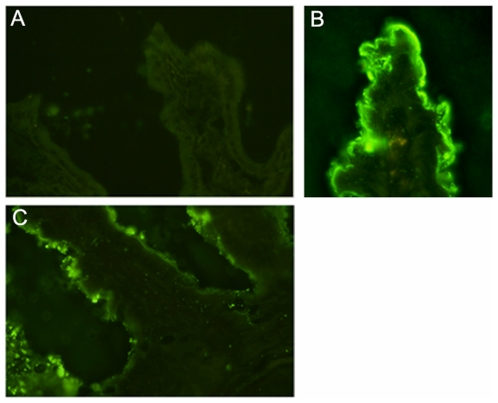
Mucosal ETX binding. Immunofluorescent detection of ETX (A) was negative in control tissues treated only with the vehicle solution. (B) ETX (2,000 LD_50_/ml) treated small intestinal segments gave a clear signal in the tips of the villi and (C) lower in the crypts.

ETX was incubated in ileal loops of mice for 20 minutes and toxin bound was analyzed by indirect immunofluorescence. ETX bound to the mucosal epithelium of small intestinal samples (Fig. 4). No fluorescence was detected in any of the control tissues analyzed. Binding was observed at the tip, center and base of the villi. However, the fluorescence intensity in the ETX small intestine treated samples was higher on the villi than in the crypts.

### Passage of molecules through the wall of the gastrointestinal tract

**Figure 5:**
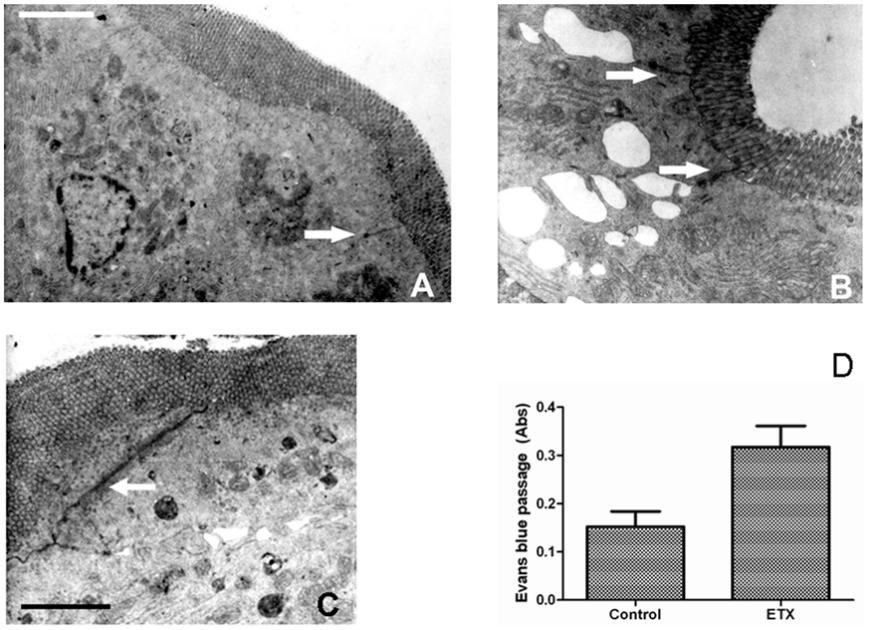
Epsilon toxin incubated in the small intestine allowed the passage of macromolecules trough the tight junction. (A) HRP infiltration in the small intestine was not observed beyond the tight junction in control loops of rats and mice. (B and C) In the luminal zone of the mucosa of ETX treated loops, HRP could be seen as strong electrondense deposits, filtering through the tight junction, from the lumen of the bowel, towards the chorion (arrows). (D) Extravasation of Evans blue bound to plasma protein was higher in ETX treated animals than control mice.

In the luminal side of the mucosa, HRP could be seen as strong electrondense deposits. In control mice given HRP intralumenally, HRP penetrated from the lumen only as far as the zonula occludens suggesting that the intestinal barrier function was well maintained (Fig. 5A). Considerable amounts of HRP were seen between intestinal epithelial cells only in mice treated with ETX, filtering through the tight junction, from the lumen of the bowel, towards the lamina propia (Fig. 5B).

The extravasation of Evans blue, which binds with plasma proteins upon intravenous injection, was examined to see whether the fluid accumulation and the changes in the intestinal permeability *in vitro* is accompanied with the leakage of plasma protein into the the intestinal lumen. Figure 5C shows the augmented extravasation of Evans blue 3 h after injection of ETX into ligated intestinal loops of mice (*P*<0.05).

### Electron microscopy analysis of small intestinal samples

**Figure 6:**
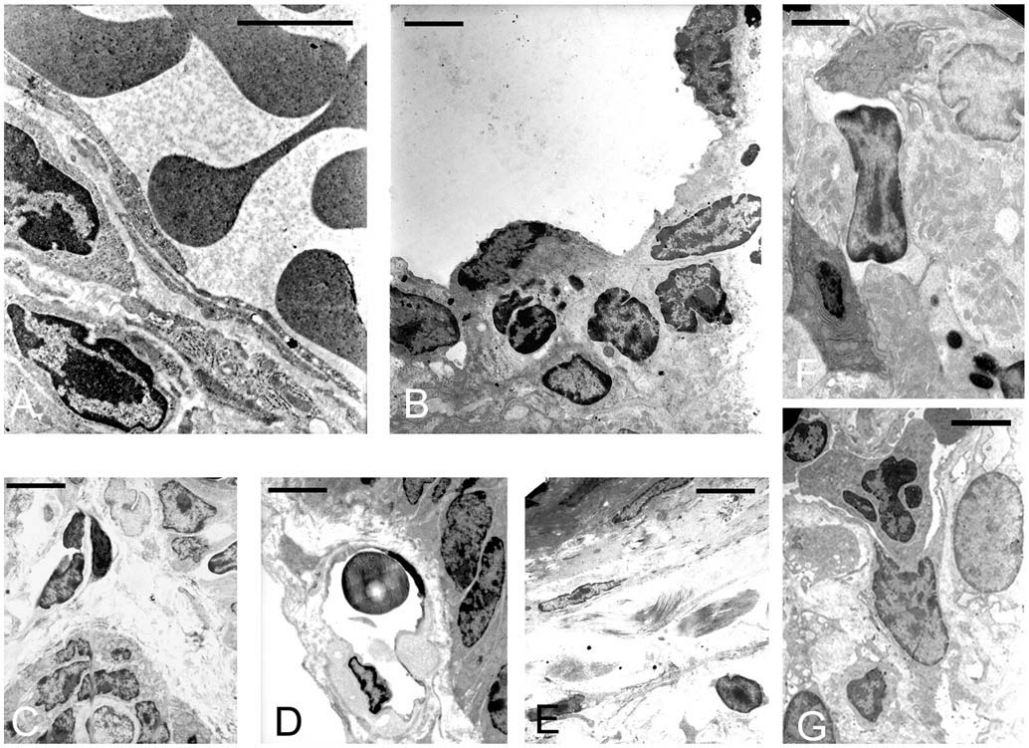
Transmission electron microscopy of control and epsilon toxin treated small intestinal loops displaying predominantly edematous changes. In the control loop (A), a blood-vessel exhibiting normal looking endothelial cells and well preserved fibroblast (scale bar = 2,5 µm). (B) In epsilon treated loops, endothelial cell displaying early degenerative changes, such as irregular nuclear shape are seen adjacent to apoptotic cells from the lamina propria (scale bar = 2 µm). (C) Platelet aggregation is seen in one vessel, in an area with evident perivascular edema and irregular-shaped cells (scale bar = 5 µm). (D) a erythrocyte in a vessel is observed surrounded by perivascular edema (scale bar = 7 µm) and (E) degenerated fibroblast with scattered collagen fibers in the edematous lamina propria are also seen (scale bar = 7 µm). (F) Plasma cell in the proximity of an apoptotic-like cell (scale bar = 4 µm) (G) and an endothelial cell surrounding a degenerative a degenerative cell.

**Figure 7:**
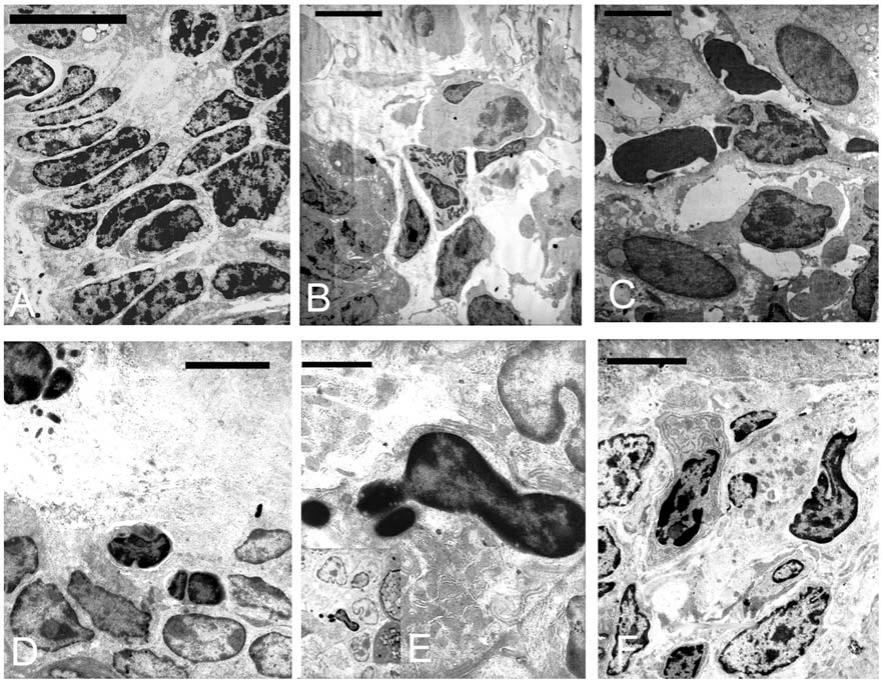
Transmission electron microscopy of control and epsilon toxin treated small intestinal loops displaying predominantly apoptotic-like changes. In control loops (A), enterocytes with normal looking nucleus and organelles are seen (scale bar = 15 µm). In epsilon treated loops (B) fibroblast with fragmented nucleus and edema with a polymorfonuclear cell (eosinophils) is seen; note as well, the electron lucent gaps between the epithelial cells (scale bar = 10 µm). (C) Abnormal looking red-blood cells together with degenerating cells, some displaying organelle and nuclear fragmentation (scale bar = 4 µm, or (D) cytoplasmatic (see in the inlet an apoptotic cell surrounded by another cells in process of nuclear fragmentation, probably at an early apoptosis stage) and (E) nuclear condensation (scale bar = 10 and 4 µm). (F) Lymphocyte in contact with a mast-cell (upper-left corner) together with some degenerating fibroblasts (scale bar = 4 µm).

No significant changes were seen in the segments analyzed of the control loops (Figs. 6A, 7A). In ETX treated loops of rats and mice, the predominant changes observed were oedema in the lamina propria (Figs. 6C–E, 7B–C), shrunken cells (Figs. 6C, 7B, D) and cellular debris (Figs. 7B, 6C). Degenerative fibroblasts (Fig. 6E) and other apoptotic-like cells were frequently seen (Figs. 7D, E), exhibiting condensed fragmented nucleus and condensed cytoplasm, sometimes containing degenerated organelles (Fig. 7E). Endothelial cells surrounding apoptotic vascular cells were frequent (Fig. 6G), and eosinophils (Fig. 7B), plasma cells and mast cells (Figs. 6F, 7F) were also seen. Some endothelial cells exhibited degenerative changes, such as irregular nuclear shape and the blood vessels were often surrounded by oedema and altered erythrocytes (Fig. 7C).

## Discussion

This study aimed to clarify the physiopathology of fluid accumulation and toxin absorption in the rodent small intestine exposed to ETX. Previous studies in ruminants (species naturally affected by ETX) contributed partially to explain the changes produced by ETX in the intestinal tract [7], [12]. However, since the impracticality to use those species in determined experimental conditions, rodents were considered as potential models since the brain lesions in sheep and mice exposed to ETX are comparable in many respects and the intravenous and intraperitoneal murine models have frequently been used to study the pathogenesis of the disease [13], [14], [15], [16], [17], [18], [19], [20]. Although to the best of our knowledge there is not information about natural infection of mice with *C. perfringens* type D, a recently developed oral challenge mouse model for studying *C. perfringens* type D infection reproduces some of the clinical findings described in sheep [6], suggesting that ETX can cross the intestinal barrier. Indeed, it has been demostrated that purified ETX can be readily absorbed from the murine small intestine into the systemic circulation [8].

The small intestine of mice incubated with ETX accumulated fluid in a concentration-dependent manner when the enteropooling assay was used. The fluid imbalance was corroborated by ileal loops and intact single-perfusion experiments. The finding suggest that ETX alters the water homeostasis of the murine small intestine, despite the absence of significant morphological change or measurable *in vitro* bioelectrical alterations, similarly as it was reported previously for ruminants [7], [12]. All of these data support the use of mice instead sheep or goats to analyse the effects of ETX by *in vivo* incubation and posterior electrophysiological *in vitro* evaluation.

The first important finding of the present study is that ETX increases the small intestinal transepithelial permeability, evidenced as reduction in the electrical resistance, in both mice and rats. Transepithelial electrical resistance can be described as resistance to the passive flow of ions as sodium and chloride. The two major pathways for such flow are through the apical and basolateral membranes in series (transcellular) and through the paracellular space. In epithelia of relatively low resistance, such as the small intestine, most passive ion flow presumably occurs through the paracellular space. This electrical resistance reduction was not due to cell damage since the epithelial cells were morphologically intact, and glucose-Na^+^ active co-transport (a mechanism of transport that occurs only when the tissue is viable) was responsive. Thus, activity of ETX resulting in decreased transepithelial resistance is likely to do so by decreasing the resistance of the paracellular pathway. As a consequence of alteration of the paracellular pathway, the intestinal mucosa becomes more permeable, and water and electrolytes (under the force of hydrostatic pressure) leak into the lumen [21]. This change in transepithelial permeability could be an important source for fluid imbalance observed in the small intestine of mice, sheep and goats. The hypothesis is that changes in water transport may arise from decreased absorption produced by increased transepithelial permeability. The augmented small intestinal permeability might be a rather slower process for fluid accumulation than intestinal secretion and could explain the fact that diarrhea or watery intestinal contents are observed during enterotoxaemia particularly if the animals survive longer than a few hours [4].

The paracellular pathway can be split into two components that may influence resistance: the tight junction and the remainder of the paracellular space. The intercellular tight junction is the rate-limiting barrier in the paracellular pathway for permeation by ions and larger solutes [22], [23]. As the rate-limiting step for paracellular transit, permeability of the tight junction defines the overall barrier function of an intact intestinal epithelium and the tight junctions have a larger conductance for cations [24]. The so-called dilution potentials are indicators for the ion selectivity of the epithelium and changes in these transepithelial potential differences reflect changes in ion selectivity of the paracellular pathway if the ion permeability of the cell membranes remains unaltered. Alteration of the dilution potential by ETX can be interpreted as ETX decreased the cation selectivity of the tight junctions.

Usually, the intestinal barrier is impermeable to microorganisms, particulate antigens and macromolecules as ETX [25]. However, oral administration of ETX produced an increased passage of immunoglobulins from the intestinal lumen to blood in mice and sheep [9] and increased lethal absorption of perfringolysin-O (protein monomer 52 kDa) orally administered to mice [10]. Also, in the present report, the instillation of ETX into ileal loops resulted in a significant increase in EB recovery when compared with vehicle-challenged loops, suggesting that the permeability of the epithelium was affected. Concordant with the electrophysiology results, it is likely that augmented EB recovery was, at least in part, the result of an increased intestinal epithelial permeability produced by ETX.

The ultrastructural analysis of epsilon treated loops demonstrated two areas of the ileum which were mostly affected: the lamina propria and the mucosa. In the lamina propria, oedema and apoptotic cell-death were the most relevant changes observed. In the mucosa, on the other hand, subtle opening of the tight junction, evidenced by the intrusion of the HRP through the junction, and oedema, evidenced as electronlucent gaps in the basal area, were the only changes noticed. It is likely that epsilon toxin is capable of opening the tight junction, increasing the mucosa permeability to macromolecules. This could enable the toxin to filter through the mucosa into the lamina propria, inducing other degenerative and inflammatory changes there. The toxin appears to alter the vascular permeability as well, thus inducing oedema. This phenomenon could enable the toxin to reach the blood stream, causing other systemic alterations.

This study also showed that the basal short-circuit current of toxin-incubated ileal mucosa *ex vivo* was increased significantly when compared with control tissues. Although the source of this ETX-induced increase in baseline *I_sc_* is not clear, it might contribute to watery intestinal contents in animals suffering the disease. Also, the addition of luminal glucose or serosal theophylline revealed significantly higher *I_sc_* stimulations in epithelia from animals pretreated with ETX in comparison with the control situation. At present it can only be speculated whether differentiation caused by ETX could account for the findings i.e. changes in the cytosolic cAMP and/or cGMP concentrations; a higher number of cells that express enhanced absorptive/secretory function; an increased density of transporters located in the apical membrane or by a faster turnover rate of each transporter; decreased receptor density or enterocyte membrane fluidity modification.

Immunoflourescence showed a marked binding of ETX to enterocytes, but it remains unknown if *in vivo* intestinal activity of ETX is a direct or an indirect effect of the toxin on epithelial cells. A few active molecules of ETX overcoming the epithelial barrier might stimulate mucosal or submucosal components which, in turn, modulate normal enterocyte function. Correspondingly, serosal application of ETX in the small intestine produced a drop of transepithelial resistance. This *in vitro* decline of electrical resistance was polar, occurring when the toxin was applicated in the basolateral rather than the apical surface of the small intestine. This polarity dependency of ETX induced biological effects may be due to either, easier access of this toxin to target molecule(s) localized on the basolateral side of the small intestine, and/or interaction with a subsequent epithelial factor. Release of mediators from these cells may conceivably affect the paracrine and/or neural regulation of the epithelium following ETX administration. The nervous system is considered a primary target of ETX: it induces damage to neurons in different brain regions [4] and affects peripheral pathways [15], [26]. Other enteric bacterial toxins are able to activate one or more components of the enteric nervous system and generate secretory responses [27]. Also, the analysis of the electron microscopy studies do not exclude pro-inflamatory changes triggered by ETX treatment or endothelial factors related with the ultrastructural changes observed in the vascular cells.

Direct damage of the endothelium produced by interaction of ETX with vascular endothelial cells increases the vessel wall permeability [28]. If ETX absorbed from the intestine causes necrosis of the endothelial cells, it could alter the intestinal vascular permeability and consequently the normal physiology of the gut. Histological analysis of the segments of small intestine exposed to ETX did not reveal damage to the endothelium, either in mice or ruminant small intestines [7], [29]. However, electron microscopy analysis revealed that endothelial cells were indeed affected after 4 h of incubation. Gardner [18] found that endothelial cells of the brain of sheep inoculated intravenously with ETX showed increased permeability to horseradish peroxidase without morphological alterations; the later were observed only after a period of 6 h had elapsed. Moreover, Morgan *et al*. [19], using horseradish peroxidase, proved that vascular leakage occurred several hours before morphological changes were evident in the brain of mice given an injection of epsilon toxin. Morphological evidence of degeneration of brain endothelial cells was observed 24 h after the intravenous inoculation of mice with *C. perfringens* epsilon toxin [20]. Therefore, it is probably that alteration of endothelial cells is associated with the physiological imbalance produced by ETX in the small intestine.

ETX is a highly lethal toxin that induces oedema and neurological damage. This toxin is responsible of enterotoxaemia in domestic animals and it is considered a potential bioterrorism agent. This study suggests that ETX alters small intestinal homeostasis and allow the paracellular passage of macromolecules as it was previously observed [9]. The increased epithelial permeability could be the key factor responsible for the absorption of lethal concentrations of ETX as well as other toxins produced during an enterotoxaemia outbreak in ruminants. Some intestinal luminal nutrients, i.e. glucose present in the small intestine during enterotoxaemia, may moderate the ETX effects on the fluid homeostasis. This study shows that ETX destabilizes enterocyte junction structures but it remains unclear if the toxin directly alters the epithelial cell or it is a later response. Future studies will explore these questions and provide further insight into the pathogenesis of ETX.

## Materials and Methods

### Animals

Conventionally reared, 20–25 g BALB-c mice and 150–200 g Wistar rats of either sex were used. The study was approved by the Animal Care and Use Committee of the University of Buenos Aires.

### Toxin

Purified epsilon prototoxin was prepared from an overnight culture of *C. perfringens* type D (strain NCTC 8346) in Trypticase-yeast-glucose medium, under anaerobic conditions at 37°C. The overnight cultures were centrifuged at 10,000 rpm for 30 min at 4°C, and the supernatant containing ETX was saved for toxin purification. The toxin was then precipitated by ammonium sulfate dialyzed against phosphate buffer solution pH 7.4 (PBS). It is referred as crude fraction. Two columns were prepared with DEAE and CM Sepharose (Pharmacia, Sweden), respectively, equilibrated in 10 mM Tris, pH 7.5. The toxin was applied to the DEAE column, and the effluent was monitored at 220 nm. The initially eluted peak was saved and applied to the CM column. Again the effluent was monitored at 220 nm, and the first peak was collected, dialyzed against PBS, and freeze-dried. Epsilon prototoxin purity was checked by SDS-PAGE. Prior to its use in these experiments, the prototoxin was reconstituted and activated by incubation at 37°C during 30 min with 0.1% trypsin (Sigma). Semi-purified *Clostridium botulinum* C2 toxin was a gift of Dr. Paticia Geogheghan.

### Enteropooling or intraluminal accumulation of fluid

Mucosal transport of fluid was determined using the enteropooling assay [30] that evaluates the net accumulation of fluid in the lumen of the small intestine [31]. After 18 h of fasting and 2 h of water deprivation, mice were treated as follow. Two groups of 6 mice were dosed orally with 1,000 LD_50_ of ETX, and sacrificed 2 and 6 h later. Other 4 groups of 6 mice were dosed orally with 0, 125, 500 or 2,000 LD_50_ of ETX in 0.5 ml of 1.5% PBS NaHCO_3_. Further groups of 6 mice were injected intravenously with 4, 0.5 or 0 LD_50_ of ETX in 0.5 ml of 1% peptone water and analyzed 3 hours later. All of these animals were sacrificed with CO_2_.

As positive control of the enteropooling assay, groups of 4 mice were injected intragastrically with 0, 12, 50 and 200 LD_50_ of C2 botulinum toxin in 0.5 ml of 1.5% PBS NaHCO_3_ and evaluated 6 hours later. C2 is a recognized enterotoxic clostridial toxin which induces fluid accumulation in the small intestine of mice [32]. Mice in negative control group were inoculated with 0.5 ml of 1.5% PBS NaHCO_3_.

The small intestine of all the mice was clamped at the pyloric sphincter and immediately before of the ileo-caecal junction and carefully removed from the abdomen. The small intestine length (L) was measured and then weighed (W1), dried of fluid and reweighed (W2). The difference between W1 and W2 divided by the length [(W1−W2)/L] shows the “enteropooling” in milligrams of fluid per centimeter of intestine and it is an indication of fluid accumulation.

### Intestinal loop test

Mice were fasted during 18 hs and deprived of water 2 h before the experiments. The mice were anesthetized by intraperitoneal injection of 0.5–0.6 g/kg of Tribromoethanol (Avertin). The abdomen was opened, and starting approximately 2 cm proximal to the ileo-cecal valve, one loop of 5-cm was isolated with cotton ligatures on the small intestine. After inoculation of 0.3 ml of PBS containing 1,000 LD_50_/ml of ETX and PBS solution as control the abdomen was closed by applying cyanoacrylate adhesive. The mice were kept under anesthesia by periodic administration of avertine until the end of the experiments, 3 h after the inoculation, when they were killed by cervical dislocation. The intestinal loops were excised, and the weight and length of each loop were measured. The net increase in the weight of the loop (in milligrams) was calculated as a relation between the weight of the loop inoculated and the length (in centimeters) of the loop.

### Perfusion Fluid and Protocol

An *in vivo* perfusion method for measuring the absorption of water by the mouse small intestine previously described was followed with slight modifications [33]. Briefly, ∼10 cm small intestinal loops were constructed as described above. One group to test ETX and one control group (4 mice each) were used. A polyethylene perfusion catheter was inserted into the gut just below the proximal ligature and secured in place by a silk ligature. A collecting cannula was inserted into the lumen of the jejunum 10 cm further down to collect the perfusion fluid. The abdominal wall was closed with three sewing points of 4–0 silk. The isolated segment of small intestine was quickly rinsed with perfusion fluid, the input catheter was attached to a perfusion pump (Minipuls II, Gilson Instruments, speed 80, Technico, producing a flow of ∼2 ml/15 min), and 15-min samples were collected from the output catheter. The perfusion fluid was a Ringer solution containing (in mM) 115 NaCl, 4 K_2_HPO_4_, 0.4 KH_2_PO_4_, 25 NaHCO_3_, 1.2 MgCl_2_, and 1.2 CaCl_2_, pH 7.4 and glucose was added at 10 mM. This solution was pre-warmed at 37°C before reach the intestinal lumen. Perfusion began with an equilibration period of 45 min; these samples were discarded. Crude fraction of ETX (500 LD_50_/ml) in Ringer solution or Ringer solution alone was perfused and after 60 minutes four 15-min samples were collected. The volume of each sample was determined by weight (assuming that 1 ml = 1 g). The actual input volume was determined before each perfusion by averaging the weight of three 15-min samples directly from the pump. The absorbed or secreted volume was calculated as collected volume minus perfused volume and expressed per centimeter of perfused bowel per minute. Fluid transport rate at time “x” was calculated as Volume _tx_–Volume _t15_. Each mouse was killed at the end of the perfusion, and the segment of jejunum was removed and its length measured.

### Bioelectric Measurements

The effects of ETX in the mouse intestinal electric parameters were analyzed *in vitro* and *ex vivo*. For *in vitro* experiments, segments of ileum were obtained from normal mice which were fasted for a minimum of 2 h and only water was provided. For *ex vivo* experiments, ileal loops were performed as described above, except that loop length was 1,5 cm and the volume injected was 0,1 ml. The mice were killed by a brief exposure to a 100% CO_2_ gas atmosphere (to induce narcosis) and a midline abdominal incision was used to excise the ileum. The excised segment was flushed of any intestinal content with cold saline solution and opened along the mesenteric border in ice cold-oxygenated Ringer solution. Each intestinal sheet (1 cm in length) without any visible Peyer's patch with a support of nylon gauze were mounted in modified Ussing chambers (Warner instruments, CA) with an exposed surface area of 0.25 cm^2^ and maintained at 37°C by water-jacketed reservoirs. The tissue sheets were independently bathed on the serosal and mucosal surfaces with 3 ml of Ringer solution. Glucose (10 mM) was included in the serosal solution and 10 mM mannitol was substituted for glucose in the mucosal bath to prevent Na^+^-coupled glucose current stimulation. Solutions were gassed with 95% O2–5% CO_2_. Transmural short circuit current (*I_sc_*, in μA/cm^2^ tissue surface area) was measured with the use of an automatic voltage clamp device (EC-800; Warner Instruments, CA) that compensates for electrode offset and the fluid resistance between the potential-measuring electrode bridges. Transepithelial potential difference (PD, in mV) was measured via a pair of Silver electrodes connected to the serosal and mucosal baths by 4% agar-Ringer (wt/vol) bridges. *I_sc_* was applied across the tissue via a pair of Ag/AgCl electrodes that were kept in contact with the serosal and mucosal baths through 4% agar-Ringer bridges. All experiments were carried out under shortcircuited conditions. Total transepithelial electrical resistance (Rt, in Ω.cm^2^ tissue surface area) was calculated by applying Ohm's law to the current deflection resulting from a 2-mV bipolar pulse across the tissue every 10 min during the course of the experiment. In all cases, the serosal side served as ground and the *I_sc_* was conventionally referred to as negative when current flowed from the lumen to the serosa.

For *in vitro* studies, the intestinal segments mounted in modified Ussing chambers were exposed to different concentration of ETX after a brief period of stabilization and monitored during 60 minutes. In *ex vivo* experiments, after the tissues achieved a stable *I_sc_* (∼30 min), the small intestine preparations were sequentially exposed to mucosal glucose (10 mM), serosal teophylline (10 mM), fresh mucosal and serosal Ringer solution, mucosal diluted Ringer solution. At the end of any experiment, glucose (10 mM) was added to the luminal bath of the small intestinal preparations as measures of tissue viability. For *in vitro* experiments, results are expresed as Δ *I_sc_* = (*I_sc_* at time t)−(*I_sc_* at time 0) and where Δ *R_t_* = (*R_t_* at time t)−(*R_t_* at time 0).

Dilution potentials were measured with modified Ringer's solution on the mucosal or serosal side of *ex vivo* treated tissues, and the data from both conditions were pooled. In the modified Ringer's solution, 70 mM NaCl were replaced by 140 mM of mannitol. Relative ion permeabilities were calculated by means of the Goldman-Hodgkin-Katz equation and partial ion conductivities were determined using the respective ion concentrations of normal Ringer's.

Rats were used to compare the *ex vivo* effects of ETX in the small intestine. The experimental protocol was essentially similar to that used with mice except that loop length was 2.5 cm and the volume injected was 0.3 ml at a concentration of 8,000 LD_50_. Intestines were incubated during 4 hours.

### Extravasation of plasma protein into the intestinal lumen

*In vivo* small intestine mucosal permeability was evaluated following a modified version of Lange's method [34]. Studies were conducted in anesthetized mice and the experimental protocol was essentialy similar to the method describe above for the intestinal loop test. After 3 h of incubation with 1,000 LD_50_ of ETX or PBS alone, a 0.1 ml of of the azo dye Evans blue (EB) in PBS (15 mg/ml) was administered intravenously. The mice were eutanized 10 minutes later by cervical dislocation. The loops were dissected, opened, and rinsed with acetylcysteine in order to remove the adherent mucus layer. Each loop was weighed and incubated with formamide for 24 hr to elute the amount of EB absorbed, which was quantitated spectrophotometrically at 560 nm.

### Histopathological analysis

Representative samples of intestinal tissues were taken immediately after the end of each experiment. Samples were immersed for 48 hs in 10% buffered formalin pH 7.2, after which they were dehydrated through graded alcohols to xylene and embedded in paraffin wax. Sections were cut at 4 µm and stained with hematoxylin and eosin and examined by light microscopy.

### Immunofluorescence detection of ETX binding to mouse intestine

ETX (2,000 LD_50_/ml in Ringer's solution) or control (Ringer's solution without ETX) was incubated during 20 minutes in intestinal loops (ileum or colon) as described above. After incubation, intestinal loops were excised and the intestinal content was flushed with Ringer's solution and tissues were immediately frozen in Tissue-Tek OCT compound (EM Science). Air-dried frozen sections of intestines were overlaid sequentially with goat polyclonal antibody anti-ETX at 1/1,000, vol/vol, and fluorescein-conjugated rabbit anti-goat (1/100, vol/vol; Sigma Aldrich Co) for 1 h each. The toxin and antibodies were diluted in PBS. Control sections were applied by ussing the same buffer but omminting the primary antibody.

### Electron microscopy

Control and treated ileum segments of mice and rats were removed immediately, flushed, cut into 0.5-cm rings, and fixed in 2% glutaraldehyde,1% paraformaldehyde in 0.1 mol/L phosphate buffer saline, pH 7.25, for 1 hour at 4°C. All of the intestines were rinsed overnight at 4°C in 0.1 mol/L 4% sucrose phosphate buffer, pH 7.5. Sections of intestinal rings 100 mm thick were cut for localization of label. For HRP experiments, the protocol was essentially similar to the method describe above for the intestinal loop test, except that after a determined period of time, loops were emptied and refilled with a HRP (mol wt 40 kDa) solution of 4 mg in 0.25 ml Ringer and incubated during 15 minutes. After fixation, demonstration of peroxidase location was achieved by incubating the sections in the dark for 20 minutes in 5 ml DAB (3, 39-diaminobenzidine) substrate medium (Sigma Immuno Chemicals, St. Louis, MO). Sections were then rinsed in Tris(hydroxymethyl) aminomethane buffer and postfixed in 1% sodium cacodylate buffered osmium tetroxide for 60 minutes. After dehydration with ethanol, the sections were treated with propylene oxide and embedded in Epon polybed 812 (Poly/Bed; Polysciences, Warrington, PA). The samples were first assessed by lightmicroscopy with blue toluidine to select the areas for TEM studies. Ultrathin sections were cut from selected areas [35]. Ultrathin sections were contrasted with 1% OsO4 and 1% uranyl acetate, dehydrated and flatembedded in Durcupan. The sections were contrasted with lead citrate, examined and photographed on a Zeiss 109 electron microscope.

### Statistical analysis

Statistical analysis was carried out using Statistix software (version 2.0). One-way analysis of variance (ANOVA) and *t*-test was used and assumed significance for a *P* value of <0.05. Throughout, descriptive statistics are reported as the mean±the standard error of the mean (SEM).
