# Correction: *Clostridium perfringens* Epsilon Toxin Increases the Small Intestinal Permeability in Mice and Rats

**DOI:** 10.1371/annotation/80ebddbb-4dda-4785-a5f2-ffd65ec1c79b

**Published:** 2013-05-15

**Authors:** Jorge Goldstein, Winston E. Morris, César Fabián Loidl, Carla Tironi-Farinati, Bruce A. McClane, Francisco A. Uzal, Mariano E. Fernandez Miyakawa

The correct name of the fourth author is: Carla Tironi-Farinati

The correct citation is: Goldstein J, Morris WE, Loidl CF, Tironi-Farinati C, McClane BA, et al. (2009) Clostridium perfringens Epsilon Toxin Increases the Small Intestinal Permeability in Mice and Rats. PLoS ONE 4(9): e7065. doi:10.1371/journal.pone.0007065 

